# The place of subjective emptiness in the structure of personality

**DOI:** 10.1186/s40479-024-00274-z

**Published:** 2024-12-04

**Authors:** Christopher J. Hopwood, Julija Gjorgjieva

**Affiliations:** University of Zurich, Zurich Switzerland, Binzmühlestrasse 14, 8050 Zurich, Switzerland

**Keywords:** Emptiness, Borderline, Personality, Interpersonal Circumplex, Diagnosis, Dimensional

## Abstract

**Background:**

Subjective emptiness is a transdiagnostic clinical dimension related to suicide, distress, and other maladaptive outcomes. Although it is typically conceptualized as a symptom of borderline personality disorder, it has a different pattern of correlates than other symptoms of that phenotype and is present in individuals with other diagnoses. The goals of this study were to replicate and extend previous findings regarding the place of emptiness as a standalone construct within a relatively comprehensive array of personality features.

**Method:**

A sample of 992 anonymous participants (M = 45.37 years, SD = 16.53) who were census-matched to the US population completed an online survey including measures of emptiness, normal-range and maladaptive trait domains and aspects, as well as interpersonal values and problems.

**Results:**

Overall, the pattern of correlations suggests that people who report feelings of emptiness tend to have negative emotions, be socially withdrawn, and are low in energy.

**Conclusion:**

These findings highlight the relevance of subjective emptiness beyond the borderline category, replicate previous findings regarding the personality and psychopathology correlates of subjective emptiness, and portray a particular profile of personality that represents a risk factor for the experience of subjective emptiness.

## Introduction

The experience of subjective emptiness is related to severe maladaptive behavior and psychiatric problems such as suicidality and mood dysregulation. So far, research on emptiness has typically been conducted in the context of Borderline Personality Disorder (BPD) [19, 20]. However, an emerging body of evidence suggests that the emptiness is relevant across disorder categories, including for people who may not have a BPD diagnosis. Price and colleagues (2022) recently developed the Subjective Emptiness Scale (SES) to study subjective emptiness as a stand-alone construct without ties to any particular diagnosis [30]. This initial validation study and follow-up studies by Konjusha et al. [20] and D’Agostino et al. [6] indicated that subjective emptiness as measured by the SES is related to maladaptive personality traits including negative affectivity, detachment, disinhibition, and psychoticism. The goal of this study was to replicate and extend these studies by examining associations between emptiness and a broad range of personality variables, including normal-range and maladaptive traits, interpersonal problems, and interpersonal values, in order to provide a relatively comprehensive picture of the personality correlates of the experience of subjective emptiness.

### Subjective emptiness

People who report emptiness feel as though there is something important missing in their experience of themselves or their world, and this feeling tends to be very distressing [10]. This feeling is experienced by patients across many mental health problem domains [2, 9, 23, 30]. In addition to borderline personality disorder, subjective emptiness has been linked to narcissistic and antisocial personality disorders [12, 41] dissociation [31], schizophrenia [40], depression [19, 20, 38, 15] and dysthymia [36]. Subjective emptiness is also associated with a number of maladaptive outcomes. Perhaps chief among them is suicidality [2, 19, 20, 24, 30]. Blasco-Fontecilla et al. [2] found that people who had attempted suicide multiple times reported doing so to relieve their feeling of numbness and emptiness [2], and several studies reported that emptiness was the most frequently cited emotion prior to a suicide attempt or self-injury [4, 19, 34]. Conversely, people tend to also feel empty after a suicide attempt [4]. Subjective emptiness is linked to other clinically significant behaviors as well, including substance abuse and problematic sexual behavior [7, 9, 19, 25, 28, 31, 33], emotion dysregulation [14, 22] social dysfunction [9, 19, 29, 32], psychiatric hospitalization, and absenteeism at work [9, 27]. Ellison et al. [9] found that the feeling of emptiness had the most consistent negative impact on psychosocial functioning among borderline personality symptoms.

### Subjective emptiness as a transdiagnostic symptom

Because most of the research on emptiness is conducted in association with the borderline category, a broader understanding of how emptiness fits within dimensional models of personality and psychopathology is underdeveloped. Situating constructs like subjective emptiness into hierarchical dimensional models of personality and psychopathology allows for a more holistic understanding of psychological phenomena [5]. By examining a wide range of constructs, this approach can help identify which areas of personality and psychopathology are likely to be associated with subjective emptiness, which helps clinicians and researchers understand common correlates and potential risk factors or consequences.

Examining constructs across a variety of levels of personality (e.g., broad vs. narrow, normal range vs. maladaptive, traits vs. problems or values) provides further texture and nuance to the conceptualiziation of a construct. Initial research described above generally suggests that emptiness falls within the internalizing domain of personality and psychopathology. Using the Subjective Emptiness Scale (SES), Price et al. found that subjective emptiness was more strongly correlated with internalizing problems such as identity problems, depression, and low meaning in life than externalizing problems like anger and impulsivity. Among maladaptive trait facets [21], emptiness had particularly strong associations with anhedonia and depressivity and negligible correlations with attention seeking, grandiosity, and manipulativeness. This finding was replicated by D’Agostino et al. [6]. However, Konjusha et al. [20] and D’Agostino et al. found that subjective emptiness was strongly correlated with all maldaptive trait domains other than antagonism, albeit correlations were strongest for negative affectivity and detachment. Thus, there is some evidence for discriminant validity, but also some evidence that subjective emptiness is related to a wide range of clinical features.

One issue that could affect discriminant validity is the pathological nature of outcome variables examined so far. The fact that most clinical variables tend to involve some level of distress may make it difficult to detect distinct patterns with a construct such as subjective emptiness. All three studies described above focused on clinical variables such as the maladaptive traits that are used for personality disorder diagnosis. In this study, we sought to expand the network of variables that may relate to subjective emptiness, including some personality variables that focus on normal-range functioning but are known to relate to clinical pheneomena. In addition to testing preregistered hypotheses about how subjective emptiness would relate to big five maladaptive trait domains, we examined normal-range personality traits at both the domain and aspect level.

We also extended previous research focusing on traits to examine associations between subjective emptiness and interpersonal functioning. We focused on two levels of interpersonal functioning: problems and values. Problems reflect things people feel they do too often or not often enough in relation to others, and are among the most common presenting complaints in psychotherapy [16]. Values reflect the goals or motives people have in relation to other people, and the frustration of interpersonal values is thought to both precipitate and result from mental health problems [17]. Like other internal experiences, subjective emptiness can be the result of social interactions, it can affect how individuals interact with others, and it can play an important role in encounters with clinicians [3]. Thus, a better understanding of the interpersonal implications of subjective emptiness could broaden the conceptualization of this issue and provide clues about how it is likely to impact relationships [39].

### The present study

In the present preregistered (https://osf.io/e9a6w/?view_only=5892c19c71b947b584f07401d1612a95) study, we examined correlates of self-reported subjective emptiness in a census-matched community sample of US adults. Replicating previous findings we predicted strong (> 0.40) positive correlations between subjective emptiness and maladaptive negative affectivity, detachment, disinhibition, psychoticism and interpersonal distress. We explored correlations between subjective emptiness and normal range traits, interpersonal problems, and interpersonal values.

## Materials and methods

We sampled 1000 anonymous adults from the Prolific Survey platform. Participants who failed both of two attention check items (answered anything other than strongly disagree to a clearly false item or provided nonsense information for all demographic variables) or participants who answered less than 80% of the items were excluded. The remaining 992 participants matched the US population relatively well in terms of race/ethnicity (782 White, 125 Black, 62 Asian/Asian-American, 8 Mixed, and 15 reported other; 928 non-Hispanic/LatinX, 61 Hispanic/LatinX, 3 did not report). Ages ranged from 18–85 years (M = 45.37, SD = 16.53). Four hundred ninety-eight of the participants were women, 481 men, and 13 reported being non-binary or other gender. In terms of political orientation, 207 reported being very liberal, 324 were liberal, 274 were moderate, 148 were conservative, 38 were very conservative and one did not report. Data are available at https://osf.io/6p42t/?view_only=4e8f206436264eae9fa2343c19033136.

### Measures

We used the five-item *Subjective Emptiness Scale* [30] to assess subjective emptiness. Item responses range from 1 (not at all true) to 4 (very true). Sample items include “I feel empty inside”. Coefficient alpha was 0.94.

We used the brief form Personality Inventory for the DSM-5 (PID-5-BF; [1]) to assess the maladaptive trait domeins negative affectivity, detachment, antagonism, disinhibition, and psychoticism. Coefficient alphas ranged from 0.72 to 0.91.

We used the *Big Five Aspect Scales* (BFAS [8];) to assess normal range personality traits. This 100-item measure assesses two aspects per trait domain: neuroticism = volatility and withdrawal; extraversion = enthusiasm and assertiveness; agreeableness = compassion and politeness; openness/intellect = intellect and openness; conscientiousness = industriousness and orderliness. Coefficient alphas ranged from 0.77 to 0.93.

We used the Inventory of *Interpersonal Problems–Short Circumplex* (IIP-SC [35];) to assess interpersonal problems. This 32-item measure has 8 octant scales that array around the interpersonal circle. Cronbach’s alpha for the octant scales ranged from 0.66 to 0.90. The circular nature of the measure allows for the summary of correlations with external variables, in this case subjective emptiness, using the structural summary method (SSM; [13, 42]. The SSM decomposes correlations with each octant scale into four parameters: elevation, the average correlation across octants, signifies associated interpersonal distress; amplitude, the distance between the average and the peak elevation, significes interpersonal specificity; displacement, or the angle of the peak association, indicates interpersonal style; prototypicality is similarity of the observed pattern of correlations with the perfect cosine curve expected given the circular nature of the instrument. When prototypicality is low, displacement cannot be interpreted and amplitude indicates general variability of associations across octant scales rather than the distance between the average and the peak.

Finally, the *Circumplex Scales of Interpersonal Values* (CSIV [26]; was used to assess interpersonal values. Like the IIP-SC, the CSIV is a circular measure whose correlates can be decomposed using the SSM. It has 64 items distributed evenly across 8 octants. However, whereas the IIP items ask about interpersonal problems, the CSIV items ask how important it is that people behave a certain way with others. Cronbach’s alpha for the octants ranged from 0.80 to 0.87.

### Analyses

We computed Pearson correlations between subjective emptiness and big five personality traits and SSM coefficients between subjective emptiness and interpersonal circumplex variables. We used an alpha level of 0.001 to determine whether a parameter differed from 0 and 95% confidence intervals, supplemented with tests of dependent correlations, to determine whether parameters differed from one another.

## Results

Correlations between subjective emptiness and personality traits are displayed in Table 1. As predicted, correlations with negative affectivity, detachment, disinhibition, and psychoticism were all at or above 0.40. Correlations with detachment and disinhibition were somewhat stronger than for the other traits. Also as expected, there was greater discriminant validity among normal range domains. The correlation with neuroticism was stronger than for any other domain, and within that domain, the correlation with with withdrawal was significantly stronger than the correlation with volatility (z = 8.30, *p* < 0.001). There were also aspect level differences for other traits, with the exception of agreeableness. Subjective emptiness had a stronger negative correlation with industriousness than orderlineness within the conscientious domain (z = 12.79, *p* < 0.001) and a stronger negative correlation with enthusiasm than assertiveness within the extraversion domain (z = 5.87, *p* < 0.001. Within openness/intellect, subjective emptiness was negatively correlated with intellect but uncorrelated with openness, a significant difference (z = 6.34, *p* < 0.001).

**Table 1:** Correlations between personality traits and subjective emptiness

	*r*	95% CI
Maladaptive Trait Domains		
Negative affectivity	.56*	[.52, .60]
Detachment	.57*	[.53, .61]
Disinhibition	.40*	[.35, .45]
Psychoticism	.48*	[.44, .53]
Antagonism	.23*	[.17, .29]
Normal Range Trait Domains and Aspects		
Neuroticism	.59*	[.55, .63]
Volatility	.47*	[.42, .52]
Withdrawal	.63*	[.59, .67]
Agreeableness	- .20*	[- .26,—.14]
Compassion	- .17*	[- .23,—.11]
Politeness	- .17*	[- .23,—.10]
Conscientiousness	- .37*	[- .42, -. 32]
Industriousness	- .49*	[- .54,—.44]
Orderliness	- .12*	[- .18,—.06]
Extraversion	- .38*	[- .43,—.33]
Enthusiasm	- .42*	[- .47,—.36]
Assertiveness	- .25*	[- .30,—.19]
Openness/intellect	- .14*	[- .20,—.08]
Openness	.02	[- .05, .08]
Intellect	- .24*	[- .30,—.18]

^*^p < .001

SSM parameters for subjective emptiness with interpersonal problems and values are shown in Table 2. As predicted, there was a strong association between subjective emptiness and interpersonal distress (problems elevation parameter). Subjective emptiness also had a fairly prototypical interpersonal problems profile with moderate specificity and an angular displacement indicating cold-submissive problems. In contrast, the elevation parameter with the values measure was mild, but protopyicality was high and amplitude was substantial. Similar to the problems measure, the displacement value suggested that people who experience more subjective emptiness tend to value being cold and submissive in social interactions.

**Table 2:** Structural summary model paramters and 95% CIs for subjective emptiness and interpersonal problems and values

	Elevation	Amplitude	Displacement	Fit
Problems	.41 (.37, .44)	.09 (.06, .13)	207.9 (182.5, 235.6)	.75
Values	.14 (.10, .19)	.13 (.10, .17)	220.4 (203.7, 239.7)	.96

## Discussion

The goal of this study was to situate subjective emptiness within a diverse and relatively comprehensive array of personality variables to provide context for how this transdiagnostic symptom relates to individual differences in personality and psychopathology. There were five main findings. First, as predicted, subjective emptiness is most strongly related to traits associated with the internalizing spectrum of psychopathology. Second, greater discriminant validity was observed for normal-range as opposed to maladaptive traits. Third, subjective emptiness had different patterns of correlation with normal range aspects, highlighting the value of examining personality constructs that are narrower than broad domains. Fourth, as predicted, subjective emptiness is associated with significant interpersonal distress. Finally, people who report emptiness tend to be cold and submissive both in terms of their interpersonal problems and values. These results add considerable specificity to previous research on the correlates of subjective emptiness.

This study also offers a new perspective on how to think about the experience of emptiness. Most previous research has subjective emptiness as a symptom of a single disorder. However, many people who do not meet citeria for borderline personality disorder are likely to experience clinically significant emptiness. In general, such people will tend to be those who also experience other internalizing symptoms, such as negative affects and social withdrawal. They are likely to be lethargic, lacking drive, enthusiasm, and intellectual curiosity. Results from this study suggest that they may also tend to be somewhat surly and perhaps at times volatile, although correlations with disagreeable traits were somewhat stronger here than in previous studies.

Nuance was also achieved by examining different levels of personality, both in the sense that we examined both broad and narrow features but also in that we examined normal and maladaptive traits, problems, and values. We found greater discriminant validity in normal-range traits than maladaptive traits. This is likely because maladaptive traits are, themselves, more correlated with one another than normal range traits because they share variance related to non-specific distress and dysfunction. We also observed that complexity is masked at the level of broad domains, as subjective emptiness was more strongly related to one aspect of personality than the other for all but one of the big five traits domains. Finally, subjective emptiness was strongly related to the elevation parameter of the interpersonal problems circumplex, indicating that people who feel empty experience relatively more interpersonal distress. The values elevation was relatively modest, indicating that there is not a strong tendency for people who tend to feel empty to have strong feelings about how social interactions should go.

This study may have some implications for the diagnosis of subjective emptiness. It is well known that lower-order features of psychopathology, such as subjective emptiness, have an uncertain location within multidimensional models of psychopathology [11, 18]. This work, in conjunction with previous findings, suggests that subjective emptiness could be considered a symptom or feature within the internalizing domain, and in particular, the region of that domain that focusses on distress [37]. Pending future research, these findings also have some implications for clinical assessment, as patients with the personality profile depicted in this study may be at risk for subjective states of emptiness, which are known to be associated with serious clinical outcomes such as suicidality. This work also adds to the growing literature supporting the SES as a standalone measure of emptiness.

However, this study was limited by the use of a cross-sectional survey in a convenience sample in the US. Future work that samples individuals from diverse regions of the world, with varying levels of clinical problems, who vary in demographic features such as age, education, and ethnicity is needed to test the generalizability of these results. Multimethod research would be helpful for better understanding the specificity of these findings and the processes by which people who feel empty produce test scores. Likewise, longitudinal or experimental methods should be used to examine causal connections between personality traits and states and the experience of subjective emptiness. Thus far, there is relatively little empirical work on the effective treatment of emptiness as a standalone clinical experience. Given the severe distress and risks associated with emptiness, this is an important area for future research. The development of the SES for the assessment of subjective emptiness and initial validation studies, including this one, provide a strong foundation for future studies on the correlates, causes, prevention, and treatment of subjective emptiness.

## Conclusion

Subjective emptiness is a relatively common transdiagnostic symptom with potentially serious clinical correlates. This study expanded the nomological network of subjective emptiness as measured by the SES by situating it within a broad array of personality variables. People who experience subjective emptiness tend to have significant negative emotions, to be socially withdrawn, and to have low levels of energy. Future work should build on these findings to further understand this important variable.

## Data Availability

Preregistration is available at: https://osf.io/e9a6w/?view_only = 5892c19c71b947b584f07401d1612a95 Data are available at: https://osf.io/6p42t/?view_only = 4e8f206436264eae9fa2343c19033136.
